# A Molecular Modeling Study of the Hydroxyflutamide Resistance Mechanism Induced by Androgen Receptor Mutations

**DOI:** 10.3390/ijms18091823

**Published:** 2017-08-23

**Authors:** Hong-Li Liu, Hai-Yang Zhong, Tian-Qing Song, Jia-Zhong Li

**Affiliations:** 1 School of Pharmacy, Lanzhou University, Lanzhou 730000, China; liuhl14@lzu.edu.cn (H.-L.L.); songtq16@lzu.edu.cn (T.-Q.S.); 2 State Key Laboratory of Applied Organic Chemistry, Department of Chemistry, Lanzhou University, Lanzhou 730000, China; zhonghy14@lzu.edu.cn

**Keywords:** hydroxyflutamide, drug resistance, androgen receptor, molecular dynamics simulation, MM-GBSA

## Abstract

Hydroxyflutamide (HF), an active metabolite of the first generation antiandrogen flutamide, was used in clinic to treat prostate cancer targeting androgen receptor (AR). However, a drug resistance problem appears after about one year’s treatment. AR T877A is the first mutation that was found to cause a resistance problem. Then W741C_T877A and F876L_T877A mutations were also reported to cause resistance to HF, while W741C and F876L single mutations cannot. In this study, molecular dynamics (MD) simulations combined with the molecular mechanics generalized Born surface area (MM-GBSA) method have been carried out to analyze the interaction mechanism between HF and wild-type (WT)/mutant ARs. The obtained results indicate that AR helix 12 (H12) plays a pivotal role in the resistance of HF. It can affect the coactivator binding site at the activation function 2 domain (AF2, surrounded by H3, H4, and H12). When H12 closes to the AR ligand-binding domain (LBD) like a lid, the coactivator binding site can be formed to promote transcription. However, once H12 is opened to expose LBD, the coactivator binding site will be distorted, leading to invalid transcription. Moreover, per-residue free energy decomposition analyses indicate that N705, T877, and M895 are vital residues in the agonist/antagonist mechanism of HF.

## 1. Introduction

Prostate cancer (PCa) is one of the main causes of cancer death and the most commonly diagnosed cancer among men worldwide [1,2]. Plenty of studies have shown that androgen receptor (AR) is a key signaling pathway leading to the emergence of PCa [3]. Hence, androgen receptor is a vital target for the treatment of PCa [4].

Androgen receptor is a ligand-inducible hormone receptor of the nuclear receptor superfamily [5,6,7], which consists of three major functional domains: an N-terminal domain (NTD), a central DNA binding domain (DBD), and a C-terminal ligand-binding domain (LBD), which is connected to the DBD by a flexible hinge region. The activation function 2 (AF2), a hydrophobic surface composed of helix 3 (H3), helix 4 (H4), and helix 12 (H12), located in the LBD, is ligand-dependent [8]. AR is mainly located in the cytoplasm of the prostate cell, combining with heat shock proteins (HSPs) [9]. Upon binding with intrinsic androgen (testosterone or dihydrotestosterone), AR will undergo a series of conformational changes, disassociating from chaperones, dimerization, and translocating into the nucleus, where it further interacts with coactivator proteins at the AF2 site. Thus, the binding between coactivators and AR at AF2 site plays an important role in the transcriptional activities of the receptor [10], which regulates the growth and survival of prostate cells.

Thus removing or inhibiting the binding of intrinsic androgens with AR is a recognized method for the treatment of PCa. Androgen ablation, by surgical or chemical castration in combination with antiandrogen such as flutamide, bicalutamide, and enzalutamide for blocking the remaining levels of AR activity, has been the standard of care for PCa for many years [11]. Although they are very efficient at the beginning, prostate tumors will become refractory after tens of months [12,13]. AR gene mutations were thought to cause resistance to antiandrogens by converting compounds from an AR antagonist to an AR agonist [14,15].

**Figure 1:**
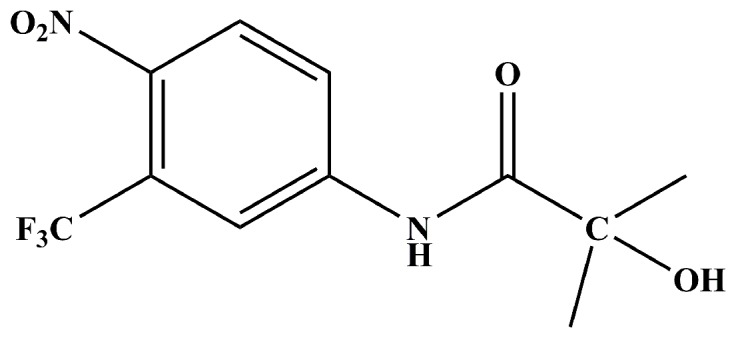
Chemical structure of hydroxyflutamide.

Hydroxyflutamide (HF) (Figure 1), an active metabolite of the first-generation antiandrogen flutamide, was used in clinic to treat PCa [16]. While HF was initially successful, Veldscholts et al. [17] reported that T877A mutation can switch HF from an AR antagonist to an agonist. Hara et al. [18] have experimentally proven that double-mutation W741C_T877A can convert HF from an AR antagonist to an AR agonist, but single-mutation W741C cannot. Balbas et al. also proved that F876L mutation would not cause a drug resistance problem [19] Furthermore, a recent study [20] has shown that F876L_T877A mutations can make hydroxyflutamide exhibit an agonist effect to some extent.

In this study, molecular dynamics (MD) simulations and molecular mechanics generalized born surface area (MM-GBSA) calculations were performed to analyze the interaction mechanisms between HF and wild-type (WT), T877A, W741C_T877A, W741C, F876L, and F876L_T877A mutant ARs. Combining the analysis of energy profiles and the binding modes of WT/mutant complexes, drug resistance mechanisms are disclosed.

## 2. Results and Discussion

### 2.1. Overall Structural Properties

**Figure 2:**
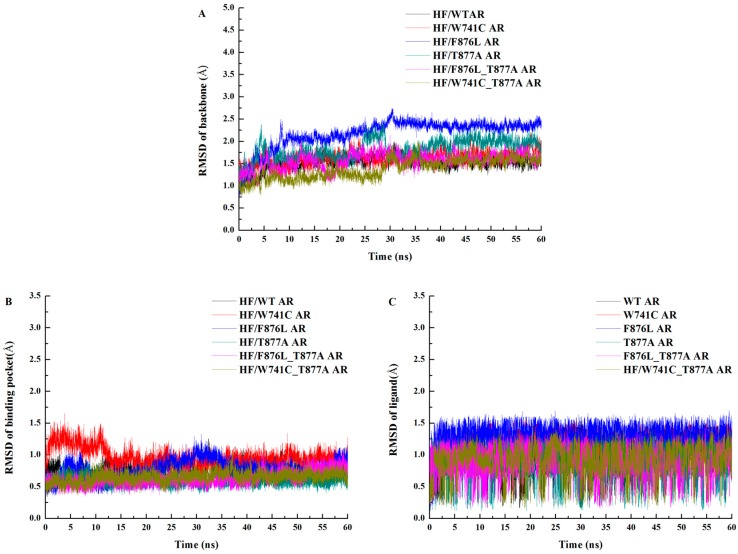
Plots of the RMSD values relative to the initial structure of wild-type and mutant ARs. (**A**) Time evolution of the RMSD of protein backbone atoms; (**B**) time evolution of the RMSD of backbone atoms for the residues around 5 Å of ligand; (**C**) time evolution of the RMSD of heavy atoms for the ligand.

The root-mean-square deviation (RMSD) of each snapshot relative to the initial structure was calculated to monitor the stability of all the studied systems (HF/WT AR, HF/T877A AR, HF/W741C AR, HF/F876L AR, HF/W741C_T877A AR, and HF/F876L_T877A AR). Figure 2 shows the RMSD of protein backbone atoms, the residues of the active site (residues within 5Å around HF) and the heavy atoms of the ligand from the initial structure. As shown in Figure 2A, the RMSD of the protein backbone atoms of all the studied systems tend to converge after 40 ns, an indication that all the systems are stable and equilibrated. Figure 2B plotted the RMSD of the residues of the active site relative to their initial structure, from which it can be seen that all the trajectories have reached convergence after 50 ns. Furthermore, the RMSD of the heavy atoms reach convergence during the simulation. Therefore, the last 10 ns of stable trajectories were used for further analysis.

**Figure 3:**
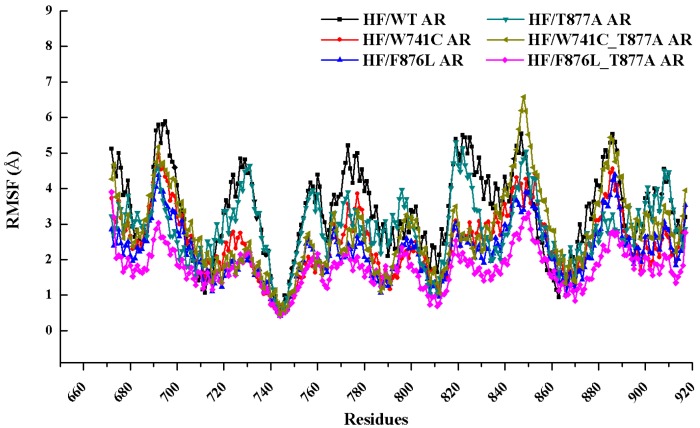
Root mean square fluctuations (RMSF) of Cα atoms relative to the initial structure for WT and mutant AR systems.

Root mean square fluctuation (RMSF) was performed to monitor the mobility of the residues around its average position during the MD trajectories. The RMSF values from the last 10 ns of MD trajectories versus the residue number for the six complexes are illustrated in Figure 3. The results indicated that the six complexes shared a similar RMSF manner of the dynamics feature. Some mutant complexes (HF/F876L_T877A AR, HF/W741C AR, and HF/F876L AR) have a smaller RMSF value than the WT system, especially in the flexible regions of (688–699, 720–732, 771–781, 817–828, 844–853, and 880–890). At the helix H3 (700–720) and H5 (740–750) regions, the RMSF values are relatively small, an indication that the active site regions of LBD are more stable than the loop regions.

**Figure 4:**
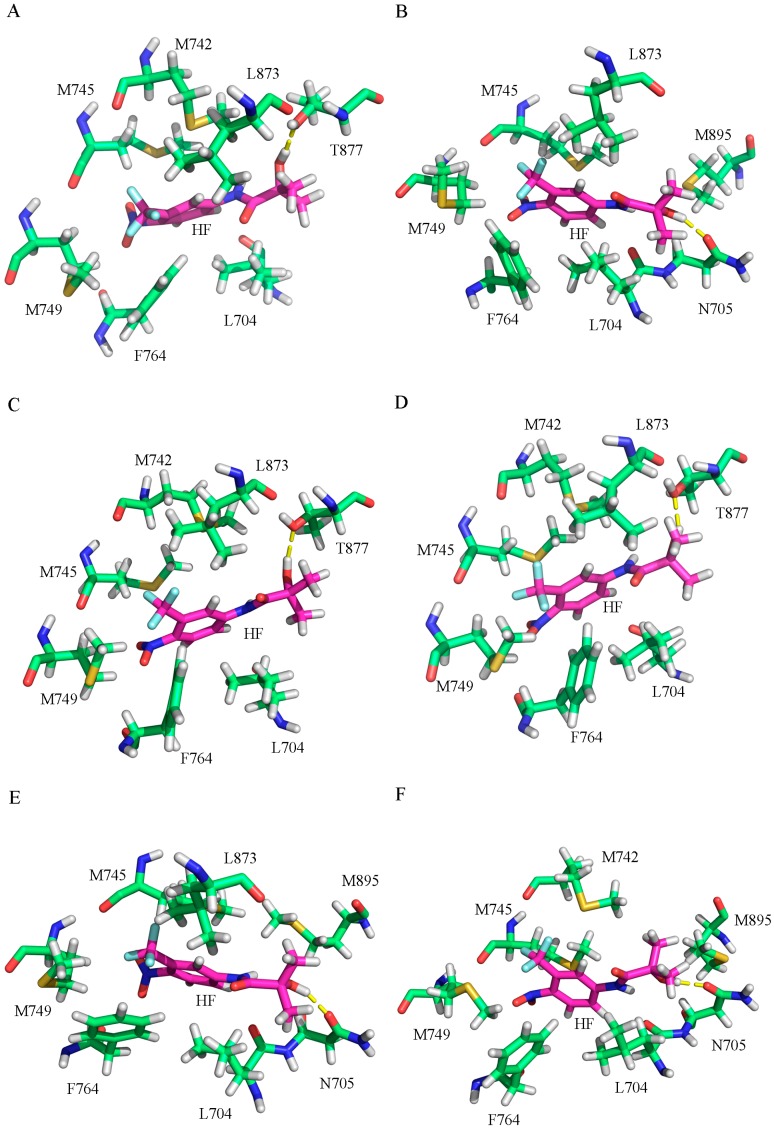
The representative structures taken from the last 10 ns of the MD simulations with the key residues of the binding pocket of the WT and mutant AR systems. (**A**) HF/WT AR, (**B**) HF/T877A AR, (**C**) HF/W741C AR, (**D**) HF/F876L AR, (**E**) HF/W741C_T877A AR, and (**F**) HF/F876L_T877A AR. For all systems, magenta sticks represent HF, yellow dashed lines represent the hydrogen bonds, and green sticks represent different residues.

Clustering analysis was performed to find the representative structures from the MD trajectory for the six systems to explore the different binding modes and interaction mechanisms between HF and WT/mutant ARs. Kclust algorithm was used for the cluster analysis with a cutoff value of 1.2 Å. The systems of HF/WT AR, HF/W741C AR, HF/F876L AR, HF/T877A AR, HF/F876L_T877A, and HF/W741C_T877A AR were clustered into three, four, three, three, three, and three classes and the representative conformation accounted for 70.68%, 51.32%, 69.94%, 46.30%, 58.38%, and 61.42%, respectively. Then, from the largest cluster, the conformation with the lowest RMSD to the cluster center was selected and shown in Figure 4. From Figure 4, we can see that the six systems have distinct interaction modes, such as dissimilar hydrogen bonds and key residues around HF, which may explain why different mutations can generate an HF resistance problem.

### 2.2. The Binding Modes of HF with T877A AR

HF is a first-generation non-steroidal antiandrogen to treat prostate cancer, and T877A mutation was the first reported mutation during the HF treatment. Veldscholts et al. [17] experimentally proved that T877A mutant AR can switch HF from an AR antagonist to an AR agonist. Here, MD simulation was performed to investigate the drug resistance mechanism caused by the T877A mutation at the molecular level.

Firstly, the MM-GBSA method was used to calculate the binding free energy, which was divided into several contribution terms (molecular mechanics, solvation energies, and entropic contribution) to explore the effect of mutation on the binding capacity of HF. Table 1 shows the averaged binding free energy over 1000 snapshots extracted evenly from the last 10 ns of MD trajectories for all the studied systems. The binding free energy values of HF/WT AR and HF/T877A AR are −6.07 kcal·mol^−1^ and −8.42 kcal·mol^−1^, respectively. The T877A mutation exhibited higher binding affinities for HF, which is consistent with the experimental results of Ozers et al. [21]. As shown in Table 1, van de Waals and electrostatic terms play important roles in the binding between HF and WT/mutant ARs.

**Figure 5:**
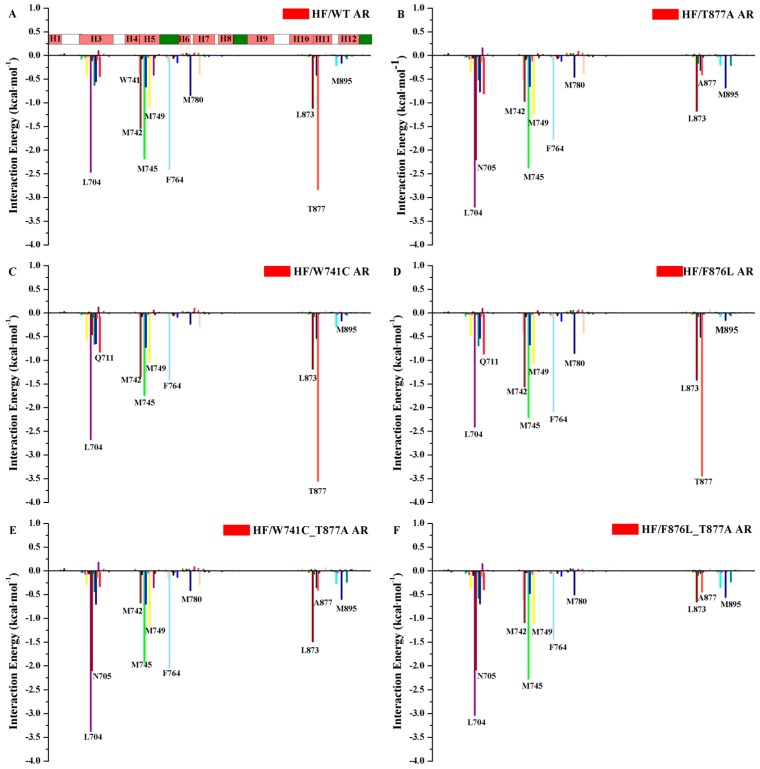
The residue contributions of WT/mutant ARs to HF binding. (**A**) HF/WT AR complex, (**B**) HF/T877A AR complex, (**C**) HF/W741C AR complex, (**D**) HF/F876L AR complex, (**E**) HF/W741C_T877A AR complex, and (**E**) HF/F876L_T877A AR complex. For the color bar, pink represents a helix, green stands for β-strand, and white is a loop region.

**Figure 6:**
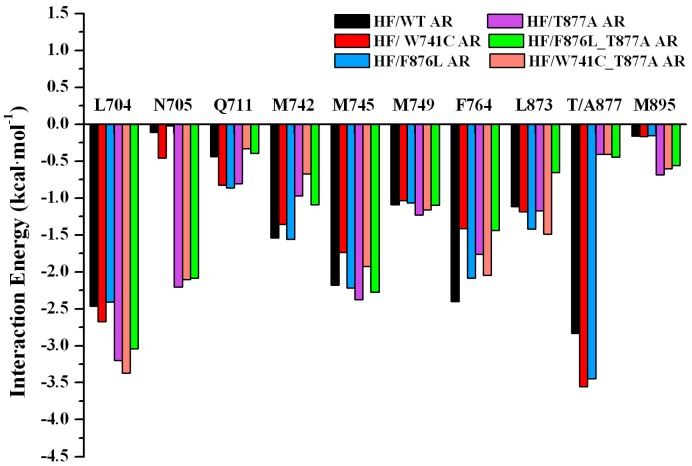
Comparison of per-residue energy decomposition for key residues for six systems: the sum of gas phase binding energy (ΔE_MM_) and the solvation free energy.

In addition, to identify the key residues related with the binding process, the total binding free energy between the protein and ligand was decomposed into each residue, and the corresponding results are depicted in Figure 5. From this figure it can be seen that the major favorable energy contributions from residues L704, M745, M749, and F764 of AR are common in all the studied systems. Compared with the HF/WT AR system, the residues located in H5–H6 make very similar contributions in all the mutant systems. The residues located in helix H12 make relatively small contributions. For the WT systems, T877 has a stronger interaction with HF, indicating that T877 plays a pivotal role in the interaction with HF. When T877 mutates from a polar threonine to a non-polar alanine, the contribution of A877 sharply dropped for the HF/T877A AR system (Figure 5B). The contributions of some key residues (L704, N705, Q711, M742, M745, M749, F764, L873, T/A877, and M895) in all systems are plotted in Figure 6. It can be seen that N705 has stronger interactions with HF in the HF/T877A AR systems compared with the WT, W741C, and F876L mutant systems. In addition, the contribution of M895 (located in H12) is somewhat increased with respect to the WT system.

**Figure 7:**
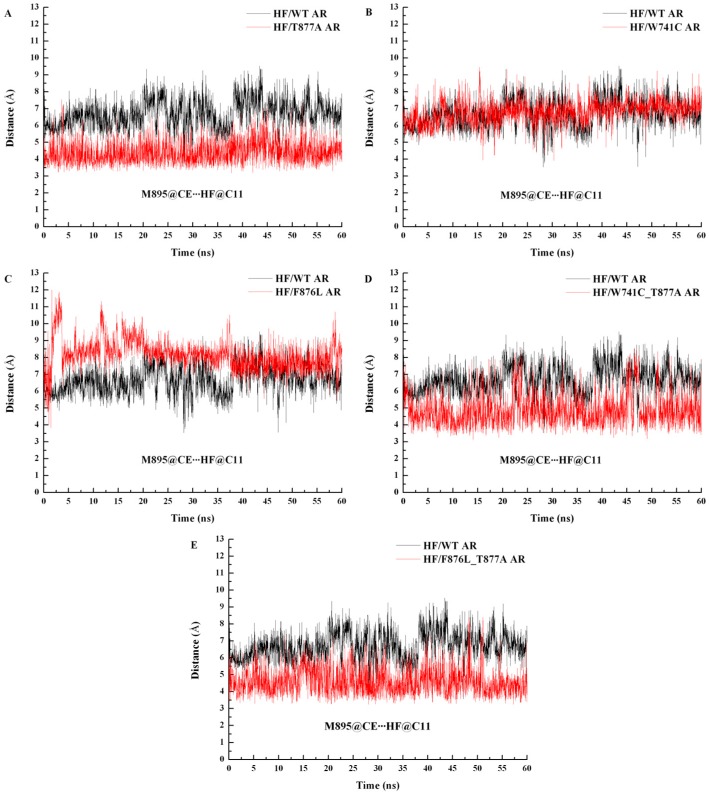
Distance of M895-HF comparison between WT AR and different mutant ARs during the all-time MD simulations. For each picture, the black line represents WT complex, and the red line is different mutant ARs. (**A**) HF/T877A AR complex, (**B**) HF/W741C AR complex, (**C**) HF/F876L AR complex, (**D**) HF/W741C_T877A AR, and (**E**) HF/F876L_T877A AR.

Furthermore, to investigate the change in M895, the distance between M895 and HF (defined by the distance of the CE atoms of residue M895 and the C11 atoms of HF) was calculated over the whole MD simulation, and the plot of distance vs. the simulation time is shown in Figure 7. From Figure 7A, it can be seen that the distance between HF and M895 in the HF/T877A AR system is significantly less than in the WT system, indicating that M895, together with H12, is near to the LBD to promote the formation of the coactivator binding site at AF2 site, which is conducive to transcription to promote the growth of prostate cancer cells. And the drug resistance problem appears.

**Figure 8:**
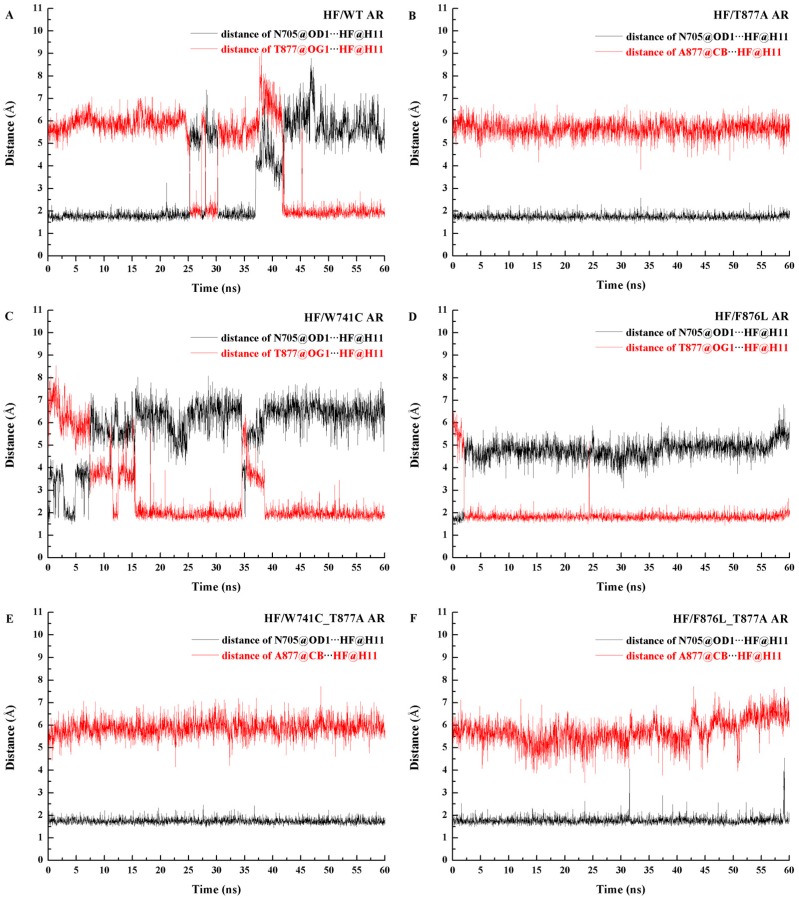
Plot of the hydrogen bond distance formed by N705-HF (black) and T/A877-HF (red). (**A**) HF/WT AR complex, (**B**) HF/T877A AR complex, (**C**) HF/W741C AR complex, (**D**) HF/F876L AR complex, (**E**) HF/W741C_T877A AR complex, and (**F**) HF/F876L_T877A AR complex during the simulations.

Hydrogen bond analysis was performed over the whole MD simulation and the last 10 ns of MD trajectories, respectively and shown in Table 2. For the HF/WT AR system, at the beginning of the simulation it formed a hydrogen bond with N705 (HF (O4-H11)∙∙∙N705 (OD1)), with 54.41% occupancy during 60 ns simulations. As the simulation continues, the hydrogen bond of HF (O4-H11)∙∙∙N705 (OD1) gradually disappears, while the hydrogen bond of HF (O4-H11)∙∙∙T877 (OG1) formed. The final 10 ns of occupancy show that the hydrogen bond of HF (O4-H11)∙∙∙T877 (OG1) is up to 99.38% while HF (O4-H11)∙∙∙N705 (OD1) disappeared. Furthermore, the distance between the hydrogen bond donor and acceptor for each system was computed and shown in Figure 8. As shown in Figure 8A, during the first 25 ns, the distance between N705 and HF is very small and then after 41 ns the hydroxyl of HF moved to T877 and formed a stable hydrogen bond with T877. For the HF/T877A AR system, when the vital polar threonine mutates to non-polar alanine in the 877 position, HF formed a stable hydrogen bond with N705 (HF (O4-H11)∙∙∙N705 (OD1), with 99.98% occupancy). Figure 8B shows that the distance between N705 and HF in the T877A mutant system is quite stable, in the range of 2 Å. Hence, this hydrogen bond was maintained well for the HF/T877A AR complex during the 60-ns simulation.

**Figure 9:**
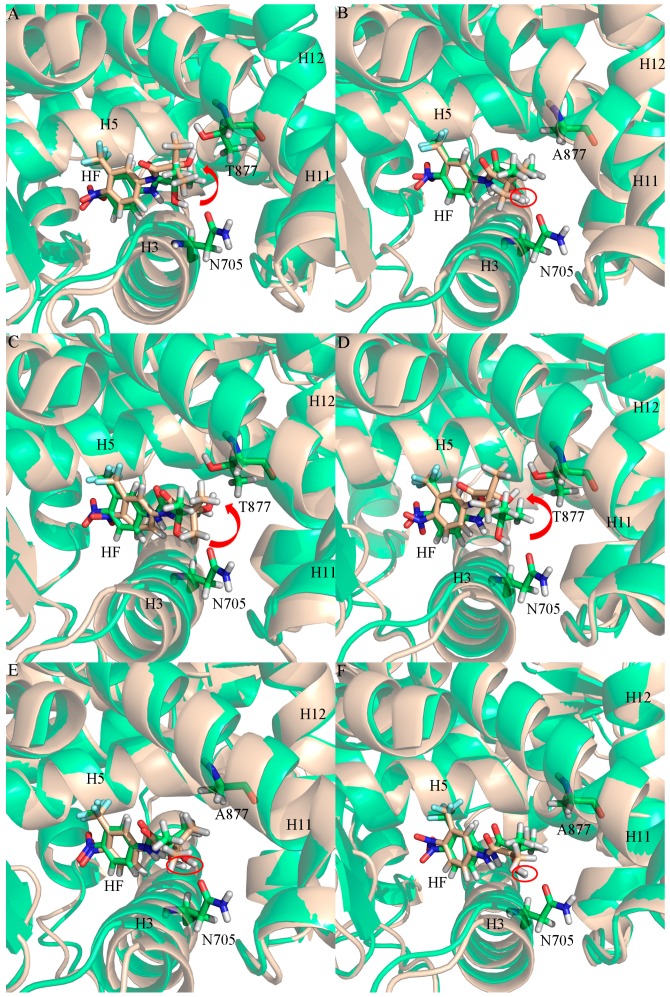
Structure comparison between initial (green) and representative structure from MD simulation (wheat). (**A**) HF/WT AR complex, (**B**) HF/T877A AR complex, (**C**) HF/W741C AR complex, (**D**) HF/F876L AR complex, (**E**) HF/W741C_T877A AR complex, and (**F**) HF/F876L_T877A AR complex.

Figure 9 shows the superimposed structure of initial and representative structure from the MD simulations. As shown in the WT complex (Figure 9A), the hydroxyl of HF in the initial structure is close to N705; when the simulation reached equilibrium, the hydroxyl group of HF oriented towards T877 and formed a stable hydrogen bond with threonine. So the residue T877 is very important to the binding between HF and WT AR. For the HF/T877A AR system (Figure 9B), the position of the hydroxyl group, close to N705, did not change in the initial and equilibrium structures.

**Figure 10:**
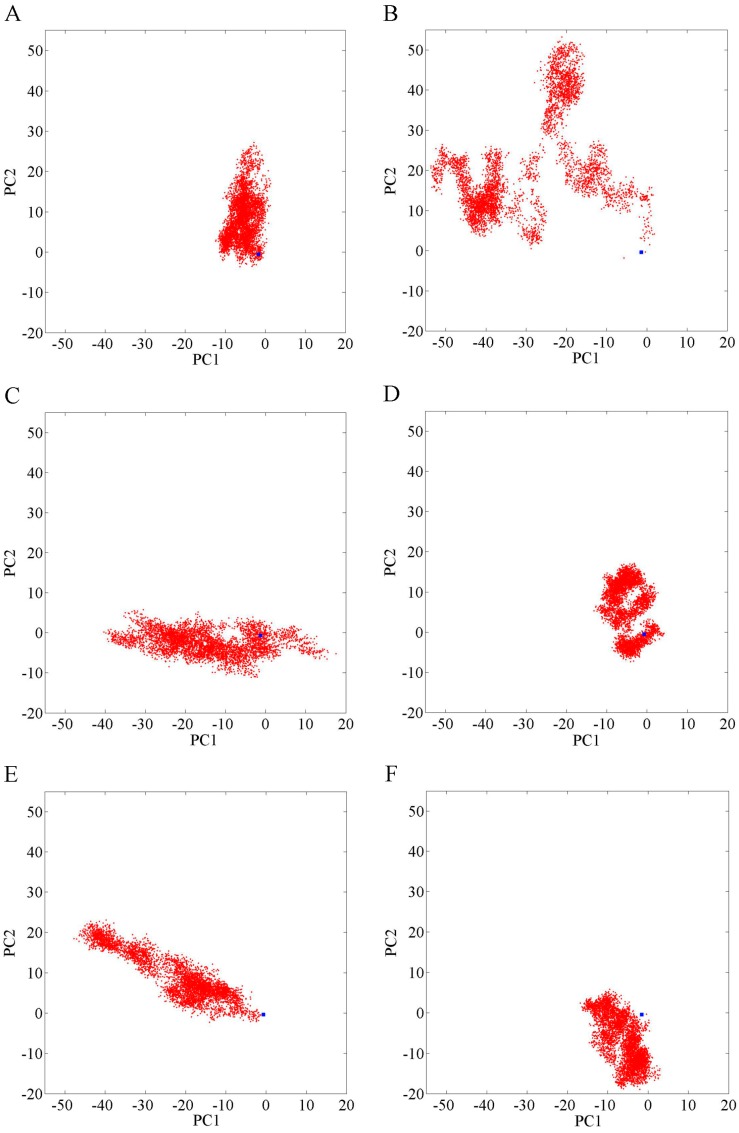
Principal components analysis of the Cα atoms in the H11_Loop_H12 motions of the simulation. The red cloud represents the last 10 ns of trajectories projected onto the first two eigenvectors for the HF/WT AR, HF/T877A AR, HF/W741C AR, HF/F876L AR, HF/W741C_T877A AR, and HF/F876L_T877A AR complexes (**A**–**F**). The blue square represents the initial structure of the transcriptionally inhibited HF/WT AR complex.

Helix H12 is a vital part of the formation of the coactivator binding AF2 site [10]. If it is pushed away from the AR LBD, the coactivator binding site will be destroyed. Principal component analysis was performed to analyze the distribution of the H11_Loop_H12 of the snapshots taken from the last 10 ns of the MD simulations. Figure 10 shows the projection of each member of the ensemble onto the plane defined by the top two eigenvectors. The red cloud represents the focus of conformation. The white area means there is no snapshot falling into this area. For the WT system (Figure 10A), there is no doubt that the blue square, which is the initial structure of the transcriptionally inhibited HF/WT AR complex, was included in the wild-type red cloud, because HF has an antagonistic effect on the wild type of AR. However, for the HF/T877A AR system (Figure 10B), the blue square is outside of the red cloud. In other words, the conformation of H11_Loop_H12 in HF/T877A AR system is not the antagonist manner but the agonist manner. Based on the structural and energetic analyses, we conclude that T877A mutation could convert HF from an AR antagonist to an agonist, which is consistent with the experimental results of Veldscholts et al. [17].

### 2.3. The Binding Modes of HF with W741C/F876L

W741C and F876L (both located in AR LBD), are the hot points of AR mutation. It is reported that W741C and F876L mutation cannot cause HF to convert from an AR antagonist to an AR agonist [18,19]. Here, MD simulations were performed to explore the resistance mechanism of W741C and F876L mutation on HF.

The binding free energy values (Table 1) of HF/W741C AR and HF/F876L AR are −2.50 kcal·mol^−1^ and −8.22 kcal·mol^−1^, respectively. The relatively small favorable van de Waals and electrostatic energies in W741C system are the main reason for its decreased binding capacity with HF. However, there is a small unfavorable polar solvation energy in the F876L mutation, so it has a relatively large binding free energy. The energy decomposition analysis shows that the residue N705 makes a smaller contribution to the W741C and F876L mutation systems, while T877 plays a vital role (as shown in Figure 6). From Figure 5C,D, it can be seen that the contributions of residues in H12 are very small, indicating that they have little interaction with HF. Figure 7 shows that the distances between M895 and HF for the W741C and F876L mutation systems are as large as around 8 Å, similar to the WT system. It is difficult for residue M895, together with H12, to cover the LBD, thus the coactivator binding site at AF2 is destroyed and leads to invalid transcription to prevent prostate cancer cell growth.

Table 2 shows that the HF/W741C AR complex formed a hydrogen bond with T877 (HF (O4-H11)∙∙∙T877 (OG1)), with 68.22% occupancy for the whole time trajectory, while the last 10 ns hydrogen bond analysis show that the occupancy of (HF (O4-H11)∙∙∙T877 (OG1)) hydrogen bond increased to as much as 99.52%, which means the hydrogen bond of HF (O4-H11)∙∙∙T877 (OG1) was formed and gradually reached stability during the simulation. The distance between T877 and HF for the HF/W741C AR system (Figure 8C) shows that T877 and HF started to form a hydrogen bond after 15 ns and reached stability after 40 ns. For the F876L mutation complex, hydrogen bond analysis of the 60 ns and last 10 ns of trajectories shows that HF formed a stable hydrogen bond with T877 (HF (O4-H11)∙∙∙T877 (OG1)) with 96.65% and 99.82% occupancy, respectively. Figure 8D shows that the distance between T877 and HF was stable at around 2.0 Å, while the distance of N705 and HF was up to 5 Å, so no hydrogen bond was formed between N705 and HF. Furthermore, the superimposition structure of initial and representative structure from MD for the W741C and F876L mutation systems (Figure 9C,D) show that the hydroxyl of the HF moved from N705 to T877 during the MD simulation and finally formed a stable hydrogen bond.

Principal component analysis results (Figure 10C,D) show that the blue square is included in the conformational distribution of H11_Loop_H12 in the W741C and F876L complexes, indicating that the androgen receptor remains an antagonistic conformation in these two systems. In other words, W741C and F876L mutations do not cause HF resistance problem. Based on the above analyses, we conclude that W741C and F876L mutations cannot switch HF from an AR antagonist to an AR agonist, in accordance with the experimental results [18,19].

### 2.4. The Binding Modes of HF with W741C_T877A/F876L_T877A

Around the ligand binding domain, there are not only single mutations, but also double mutations like W741C_T877A and F876L_T877A. Hara et al. [18] experimentally proved that W741C_T877A mutations can cause drug resistance to HF.

The binding free energies of W741C_T877A and F876L_T877A are −5.77 kcal·mol^−1^ and −4.52 kcal·mol^−1^, respectively. Their binding capacity is reduced compared to the WT AR. Although the HF/W741C_T877A AR complex has high favorable electrostatic energy, high unfavorable polar solvation energy counteracts it and reduces the binding free energy. For the HF/F876L_T877A complex, the low favorable electrostatic energies and van der Waals energies lead to the low binding free energy. The mutations affect the binding capacity between AR and HF to some extent.

Both W741C_T877A and F876L_T877A double-mutation systems contain a T877A mutation. The decomposition of free energy (Figure 6) shows that when the residue of T877 mutates to A877, the contribution of A877 is significantly reduced, while the contribution of residue N705 is increased. Although the contribution of residue M895 to the W741C_T877A and F876L_T877A systems is not large (Figure 5E,F), its contribution increased relative to the HF/WT system, which means the interaction between M895 and HF increased, similar to the T877A system. Moreover, the distance between M895 and HF for the W741C_T877A and F876L_T877A systems (shown in Figure 7D,E) are lower than for the WT complex, indicating that M895 pulls H12 to cover the LBD to play an agonistic role.

As shown in Table 2, the W741C_T877A and F876L_T877A complex formed stable hydrogen bonds with N705 (HF (O4-H11)∙∙∙N705 (OD1), with 99.98% and 99.67% occupancy for the whole time trajectory, respectively) rather than the residue T877. The free energy decomposition results also show that the contributions of N705 in these two complexes are bigger than in the HF/WT complex. Figure 8E,F show that the distance between N705 and HF is stable at around 2.0 Å, while the distance of A877 and HF is up to 6 Å. Therefore, no hydrogen bond was formed. Furthermore, as shown in Figure 9E,F, the hydroxyl of HF in the W741C_T877A and F876L_T877A systems did not move during the process of simulation, but remained in the vicinity of N705 and formed a stable hydrogen bond with it.

Principal component analysis results (Figure 10E,F) show that the blue squares are not included in the H11_Loop_H12 conformational distributions (red cloud) for the W741C_T877A and F876L_T877A complexes. That is to say that the conformation H11_Loop_H12 did not take antagonistic configuration during the MD simulation process for these two complexes, indicating that these two double mutation systems, both containing T877A, can switch HF from AR antagonist to agonist. From the above analyses we suggest that W741C_T877A and F876L_T877A mutation can cause HF to produce drug resistance [18,20]. Although single mutations of W741C and F876L cannot make HF resistant, as long as the T877A happens, this mutation will convert HF from AR antagonist to AR agonist.

## 3. Methods

### 3.1. Preparation of the Starting Structures

Atomic coordinates of the X-ray crystal structure of WT AR and HF in a complex with T877A AR were obtained from the Protein Data Bank (PDB ID: 2AXA and 2AX6) [22]. As the X-ray structures of the W741C, F876L, W741C_T877A, and F876L_T877A mutant ARs are unknown, the Swiss-Pdb Viewer (v4.1.0) [23] program was applied to generate the 3D structures of these mutant ARs by substituting specific residues using the 2AXA as template for W741C and F876L ARs, and 2AX6 as the template for W741C_T877A and F876L_T877A double mutations. HF was extracted from the T877A complex (PDB ID: 2AX6), then hydrogen atoms and Gasteiger–Hückel charge were added in SYBYL 6.9 software [24]. Finally, HF was docked with WT and mutant ARs (except T877A AR) by the CDOCKER module of Discovery Studio 2.5 software [25].

Before the MD simulations, geometric optimization and the electrostatic potential on HF were calculated at the HF/6-31G* level of Gaussian 09 suit [26]. The partial atomic charges of the HF were determined by the restrained electrostatic potential (RESP) fitting method [27,28,29]. The force field parameters of HF were created using the Antechamber program in the Amber 12 package [30] and described by the General Amber Force Field (GAFF) [31], and the protein parameters were described by a standard ff99SB force field [32]. The tleap module of the Amber 12 package was used to add all missing hydrogen atoms of the proteins. Then, an appropriate number of chloride counterions was added to maintain the electro-neutrality of these systems, and each system was immersed into a cubic box of TIP3P [33] water molecules with at least 10 Å distance around the complex.

### 3.2. Molecular Dynamics Simulations

In this work, all the MD simulations were carried out using the AMBER 12 package. The SHAKE algorithm was used to restrain bond lengths involving hydrogen atoms [34]. During the simulations, periodic boundary conditions were employed and all electrostatic interactions were calculated using the particle mesh Ewald (PME) [35,36] method. For all MD simulations, the time-step was set to 2 fs and non-bonded cutoff was set to 10 Å. The energy minimization, heating and equilibration protocols of the studied complexes were carried out using the Sander module. Initially, to remove the bad contacts between the solute and solvent, three stepwise rounds of minimization were performed. Firstly, all solutes were constrained to minimize all the water molecules by a harmonic constraint potential with 5 kcal·mol^−1^·Å^−2^. Then, backbone atoms of the protein were constrained with a 3.0 kcal·mol^−1^·Å^−2^ force constant, which enables the amino acid side chains to find a better way to hold the HF. Afterward, all the atoms were allowed to move freely without any restraint. Each round consists of 2500 cycles of steepest descent minimization followed by 2500 cycles of conjugated gradient minimization. After minimization, these systems were heated from 0 to 310.0 K over a period of 50 ps in the canonical (NVT) ensemble using a Langevin thermostat with a coupling coefficient of 2.0 ps^−1^ and a harmonic restraint force of 2.0 kcal·mol^−1^·Å^−2^. Subsequently, isothermal isobaric (NPT) ensemble was used to adjust the solvent density to equilibrium by restraining all the atoms of the solute with the harmonic restraint force of 1.0 kcal·mol^−1^·Å^−2^ over 50 ps. Moreover, an additional equilibration of 1 ns in the NPT ensemble without any restraint was performed. Finally, 60 ns MD simulations were performed by PMEMD module without any restraints in the NPT ensemble at a temperature of 310.0 K and a pressure of 1 atm. Coordinate trajectory was recorded every 2 ps and the temperature was regulated using a Langein thermostat.

### 3.3. Binding Free Energy Calculations

The binding free energies (ΔG_bind_) between protein and ligand were calculated using the MM-GBSA method [37,38,39], which was implemented in the AMBER 12 program. One thousand snapshots from the last 10 ns stable MD production trajectory of the complex with an interval of 10 ps were generated. For each snapshot, free energy was calculated for each molecular species (complex, receptor and ligand), and the binding free energy is estimated as follows:

ΔG_bind_ = G_complex_ − (G_protein_ + G_ligand_) (1)

where G_complex_, G_protein_ and G_ligand_ are the free energy of complex, receptor and ligand, respectively. The binding free energy (ΔG_bind_) is evaluated by a sum of the changes in gas phase binding energy (ΔE_MM_), the solvation free energy (ΔG_sol_) and entropic (TΔS) contribution.

ΔG_bind_ = ΔE_MM_ + ΔG_sol_ − TΔS (2)

ΔE_MM_ = ΔE_int_ + ΔE_ele_ + ΔE_vdw_ (3)

ΔG_sol_ = ΔG_p_ + ΔG_np_ (4)

ΔG_np_ = γSASA + β (5)

ΔE_MM_ is further divided into internal energy (ΔE_int_) of bond, angles, and torsions, van der Waals (ΔE_vdw_), and electrostatic energies (ΔE_ele_). The solvation free energy (ΔG_sol_) is further divided into polar (ΔG_p_) and nonpolar (ΔG_np_) components. The polar component was calculated with a GBSA module of the AMBER 12 suit. Dielectric constants of 1.0 and 80.0 were used for solute and solvent. The nonpolar component was determined by the solvent-accessible surface area (SASA) with a probe radius of 1.4 Å [40]. The values of γ and β, which are empirical constants, were set to 0.0072 kcal·mol^−1^·Å^−2^ and 0, respectively [41].

The conformational entropy contributions to the binding free energy were estimated for 50 snapshots, which were extracted from the last 10 ns of MD trajectories with 200 ps time intervals, using normal mode analysis with the AMBER 12 NMODE program [42].

### 3.4. Energy Decomposition

A residue energy decomposition analysis was performed with the MM-GBSA method to obtain the contribution of each residue to the total binding free energy. The binding interaction of each HF–residue pair includes molecular mechanics energies and solvation energies without considering the contribution of entropies.

### 3.5. Principal Component Analysis

Principal component (PC) analysis is an important tool for investigating the conformation change of proteins. In this study, the distribution of the H11_Loop_H12 conformations was analyzed with the period of last 10 ns trajectory. It is based on the covariance matrix [43]:

σ*_ij_* = <(d*_i_*- < d*_i_*>)(d*_j_*- < d*_j_*>)>, (6)

where d_1_, … d_36_ are the distances from the Cα of residues (residues 873–908) in H11_Loop_H12 to the mass center of the core structure (residues 698–844) and the angle bracket represents the average over all sampled conformations. The matrix σ*_ij_* was symmetrical and could be diagonalized to obtain eigenvectors γ (PC1, PC2, … … PCn) and eigenvalues λn. The γ are arranged in a descending order. The first few eigenvectors contain a major percentage of the conformation distribution, so PC1 and PC2 are sufficient to describe the distribution of the conformation.

### 3.6. MD Trajectory Analysis

The “ptraj” module of the Amber 12 software was used to analyze the MD simulation trajectories. The root mean square deviation (RMSD) was calculated to monitor the convergence of all the studied systems. The root mean square fluctuation (RMSF) about the mean position of atoms was calculated only for Cα. The hydrogen bond criterion used was an acceptor–donor distance of <0.35 nm and acceptor…H-donor angle >120°. Then 60-ns trajectories and the last 10 ns stable trajectories were used to calculate the hydrogen bond. The MMTSB toolset was employed to calculate the most populated conformations through a cluster analysis based on the kclust algorithm [44]. Then, from the largest cluster, the conformation with the lowest RMSD to the cluster center was selected.

## 4. Conclusions

In this study, MD simulations in conjunction with free energy analysis using the MM-GBSA method were used to investigate how different mutations cause HF to be switched from an AR antagonist to an AR agonist. The free energy decomposition analyses show that residues L704, N705, Q711, M742, M745, M749, F764, L873, T877, and M895 play important roles in the interaction between HF and WT/mutant ARs. Especially T877: when it mutates to A877, its contribution will be significantly reduced but the contributions of N705 and M895 increased, such as in the T877A, F876L_T877A, and W741C_T877A complexes. Moreover, the distances between M895 and HF are relatively small in these complexes, indicating that M895, together with H12, is near the AR LBD to promote the formation of a coactivator binding site at AF2, which is conducive to transcription to promote prostate cancer cell growth. The hydrogen bond analysis shows that T877 formed stable hydrogen bond with HF for the WT, W741C, and F876L complexes. However, when T877 mutates to A877, the hydroxyl of HF will form a strong hydrogen bond with N705 and not move to A877. Furthermore, principal component analysis implies that the conformation distributions of H11_Loop_H12 in the WT, W741C, and F876L complexes contain the initial structure of the transcriptionally inhibited form (blue square), but the systems of T877A, W741C_T877A, and F876L_T877A did not contain the blue square. So we conclude that W741C and F876L mutations cannot convert HF from an AR antagonist to an AR agonist, but T877A, W741C_T877A, and F876L_T877A mutations can cause an HF resistance problem. The information obtained from this study may be valuable for future rational design of a novel AR antagonist. 

## Figures and Tables

**Table 1:** The calculated binding free energies (kcal·mol^−1^) and the individual energy components for the WT/mutant systems.

Complexes	Contribution						
	ΔE_ele_	ΔE_vdw_	ΔG_p_	ΔG_np_	ΔE_bind_	−TΔS	ΔG_bind_
HF/WT AR	−28.21	−40.24	44.92	−5.66	−29.19	23.12	−6.07
HF/T877A AR	−32.34	−40.16	47.45	−5.63	−30.67	22.25	−8.42
HF/W741C AR	−23.22	−38.36	41.52	−5.53	−25.60	23.10	−2.50
HF/F876L AR	−23.38	−39.45	39.42	−5.63	−29.04	20.82	−8.22
HF/W741C_T877A AR	−36.67	−39.89	53.16	−5.67	−29.08	23.12	−5.77
HF/F876L_T877A AR	−24.34	−35.36	39.47	−5.19	−25.31	20.79	−4.52

**Table 2:** The hydrogen bonds between HF and key residues in ARs.

Complex	Interaction Pair	Distance (Å) *		Angle (°) *		Occupancy (%)	
		60 ns	10 ns	60 ns	10 ns	60 ns	10 ns
HF/WT AR	HF@O4-H11∙∙∙N705 (OD1)	2.71	-	159.15	-	54.41	-
	HF@O4-H11∙∙∙T877 (OG1)	2.87	2.87	161.89	162.05	36.78	99.38
HF/T877A AR	HF@O4-H11∙∙∙N705 (OD1)	2.68	2.68	160.73	160.34	99.98	99.98
HF/W741C AR	HF@O4-H11∙∙∙T877 (OG1)	2.89	2.86	163.49	163.88	68.22	99.52
HF/F876L AR	HF@O4-H11∙∙∙T877 (OG1)	2.75	2.77	162.15	162.13	96.65	99.82
HF/W741C_T877A AR	HF@O4-H11∙∙∙N705 (OD1)	2.67	2.67	161.27	161.54	99.98	99.96
HF/F876L_T877A AR	HF@O4-H11∙∙∙N705 (OD1)	2.70	2.71	160.91	160.44	99.67	98.56

* The hydrogen bonds are determined by the acceptor…donor atom distance of <0.35 nm and acceptor…H-donor angle of >120°.
